# High-temporal resolution (<6 ms) Cine Steady-State Free Precession (SSFP) imaging for assessing LV diastolic function

**DOI:** 10.1186/1532-429X-11-S1-P74

**Published:** 2009-01-28

**Authors:** Ramkumar Krishnamurthy, Benjamin Cheong, Amol Pednekar, Raja Muthupillai

**Affiliations:** 1 Rice University, Houston, TX USA; 2 St. Luke's Episcopal Hospital, Houston, TX USA; 3 Philips Medical Systems, Houston, TX USA

**Keywords:** Temporal Resolution, Aortic Valve Closure, Peak Ejection Rate, Apical Slice, Cine SSFP

## Background

While a modest temporal resolution of conventional cine SSFP MRI (30–50 ms) is sufficient for calculating parameters characterizing LV systolic function, e.g., stroke volume (SV) or ejection fraction (EF), it is insufficient for characterizing diastolic relaxation phenomena via metrics such as isovolumic relaxation time (IVRT) or Time to Peak Filling Rate (TPFR).

## Purpose

We describe a fast cine MR imaging technique with a temporal resolution of under 6 ms using the acceleration techniques of Sensitivity Encoding (SENSE) and spatial-temporal frequency Broad-use Linear Acquisition Speed-up Technique (k-t BLAST).

## Methods

### MR acquisition

High temporal resolution cine SSFP images were acquired in 13 normal volunteers (12 m, 35 ± 8 years) using 32 channel/16 channel cardiac coils at 1.5 T (Achieva, Philips Medical Systems) using SENSE and k-t BLAST. The acquisition parameters were: acquired voxel size: 2 × 2 × 8 mm^3^, temporal resolution 5.8–6 ms; TR/TE/flip = 2.8–3 ms/1.4–1.5 ms/55°; breath-hold time : 18 heart-beats/slice. The effective acceleration factors were: SENSE – 3; k-t BLAST – 3.8.

Images were acquired along short-axis (basal, mid-cavity and apical) and long-axis (LVOT and 4-chamber) orientations. All subjects underwent echo immediately after MR.

### Data analysis

The LV cavity was segmented from cine MR images using a custom-built algorithm, and the time-volume (T-V) curves were generated. From the T-V curve, diastolic parameters IVRT, PFR, and TPFR were determined.

## Results

**Figure 1:**
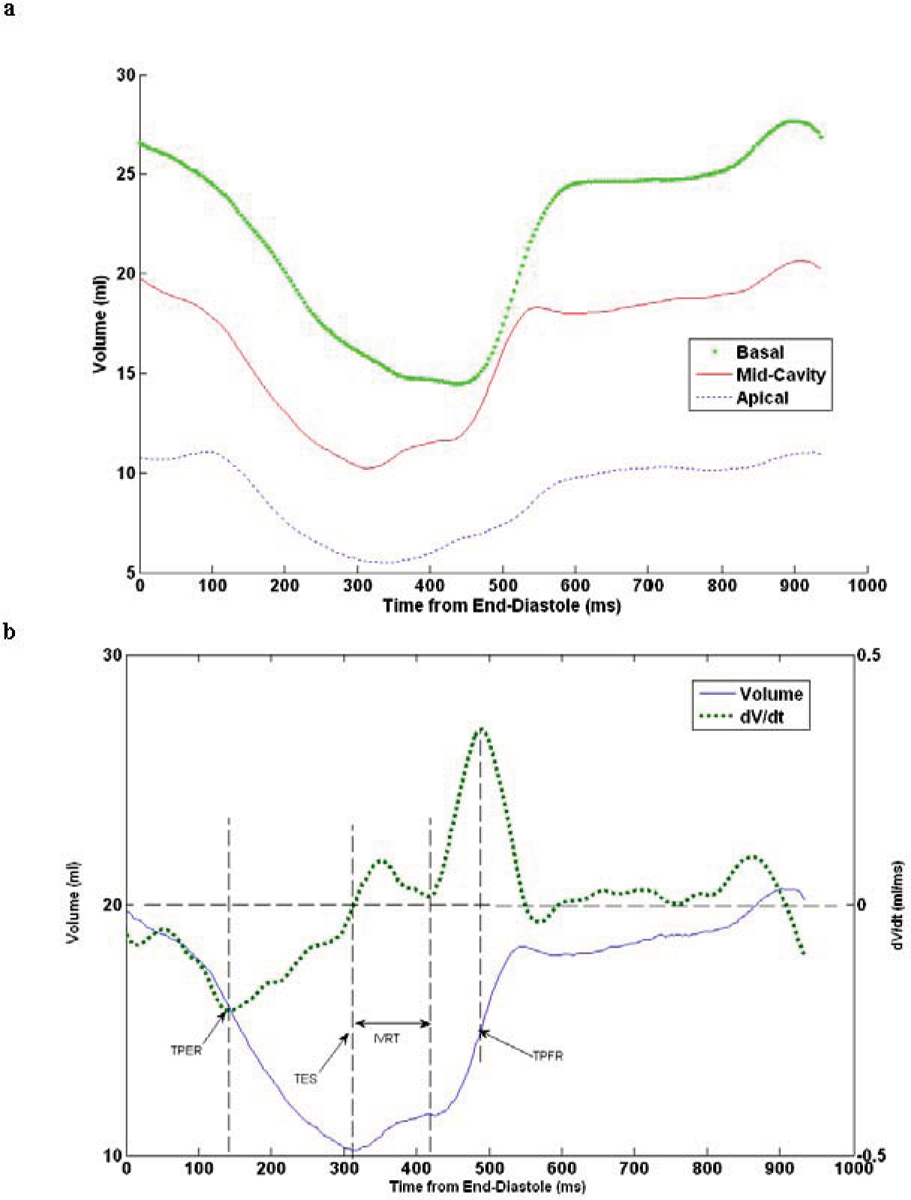
**a) Volume-time curves of LV short axis slices at different locations**. B) Time-Volume and dV/dt curves of mid-LV cavity short axis slice.

Representative T-V curves from the basal, mid, and apical LV slices are in Figure 1a. All relaxation metrics were calculated from the T-V curve of the mid LV cavity (Figure 1b). The iso-volumic period is marked by local minima (dip) in the dV/dt curve immediately after the occurrence of Time to End Systole (TES) (zero-crossing in dV/dt). The TPFR and Time to Peak Ejection Rate (TPER) values are obtained as the time of maxima and minima of dV/dt values (Figure 1b).

**Figure 2:**
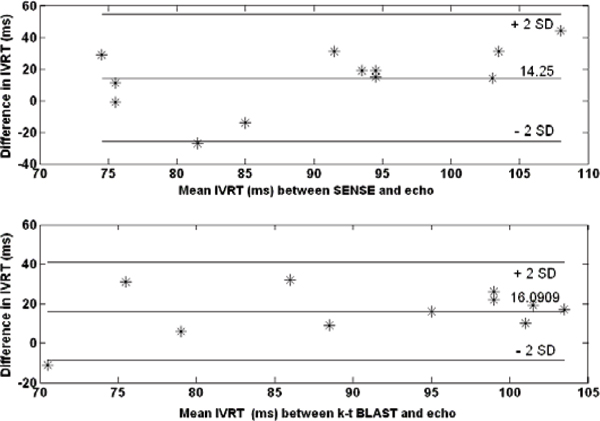
**Bland-Altman analysis of IVRT between MRI and echo values**.

The Bland Altman analysis revealed close agreement in the values of TES, IVRT and TPFR between kt-BLAST and SENSE acquisitions (Table 1). MR estimate of IVRT based on T-V curves was consistently higher (about 15 ms) compared to echocardiography (Figure 2).

**Table 1:** Bland-Altman Analysis: k-t BLAST and SENSE cine SSFP

	Bias ± SD (ms)	Mean (ms)	Bias ± SD (%)
TES	2.32 ± 18.1	296.2	0.76 ± 6.1
TPFR	5.8 ± 8.7	480.0	1.2 ± 1.8
IVRT	-0.08 ± 16.6	99.7	-0.08 ± 16.6

## Discussion

Firstly, as shown in Figure 1a, the filling patterns at the basal, mid, and apical slices are different. The dV/dt curve of the mid-LV cavity (figure 1b) had a marked dip after TES, highlighting the region of IVRT. This feature was not always present in the basal and apical filling patterns across subjects. We hypothesize that this may be attributed to more pronounced shape changes in the base and apex of the LV compared to the mid-LV. A similar observation has also been made by Zwanenburg et al. who noted that the maximum radial strain of the mid LV cavity coincided more with aortic valve closure than for basal or apical slices[1].

Secondly, our results show that MRI estimates of IVRT are consistently higher by about 15 ms compared to echo. Unlike echo, MRI estimates use the LV volume curves, and as a result, the calculation of IVRT using MRI may also include the proto-diastolic period.

Lastly, the ability to obtain high-temporal resolution cine MR images also allows for the estimation of hitherto unexploited parameters such as PFR, TPFR, PER, and TPER and can pave the way for a more comprehensive analysis of LV function.

## Conclusion

1. Cine SSFP images with a temporal resolution of 5–6 ms yield time-volume curves that can be used to estimate diastolic functional parameters such as TPFR, PFR, and IVRT.

2. Diastolic indices computed from high-temporal resolution k-t BLAST and SENSE are in agreement.

3. IVRT estimated from MRI time-volume curves is longer than echocardiography (around 14–16 ms).
